# Prognostic and Clinical Significance of COX-2 Overexpression in Laryngeal Cancer: A Meta-Analysis

**DOI:** 10.3389/fonc.2022.854946

**Published:** 2022-04-22

**Authors:** Jingwei Du, Jun Feng, Deyan Luo, Lijuan Peng

**Affiliations:** ^1^Department of Otolaryngology-Head and Neck Surgery, Nanchong Central Hospital, The Second Clinical Medical College, North Sichuan Medical College, Nanchong, China; ^2^Department of Pathogen Biology, School of Basic Medical Sciences & Forensic Medicine, North Sichuan Medical College, Nanchong, China

**Keywords:** COX-2, expression, laryngeal cancer, meta-analysis, prognosis

## Abstract

**Objective:**

Several studies were conducted to explore the clinical significance of cyclooxygenase-2 (COX-2) overexpression in laryngeal cancer. However, the associations between COX-2 overexpression and clinicopathological characteristics of laryngeal cancer patients remained unclear. Here, we performed a meta-analysis to eva-TY -40luate the role of COX-2 overexpression in the risk, clinical progression, and progno\sis of laryngeal cancer.

**Methods:**

The eligible literature was obtained from PubMed, Embase, Web of Science, and China National Knowledge Infrastructure (CNKI) databases. Odds ratio (OR), risk ratio (RR), and 95% confidence interval (CI) were calculated to assess the strength of the associations, and *I*^2^ statistics were used to evaluate heterogeneity among studies. Publication bias was detected with Begg’s test and Egger’s test.

**Results:**

A total of 47 eligible articles were included for the meta-analysis after screening. COX-2 expression levels in the laryngeal cancer patients were significantly higher than those in the normal controls (OR = 11.62, 95% CI: 6.96–19.40, *P* < 0.05). The pooled results also showed that there were significant correlations between COX-2 overexpression and clinicopathological characteristics (tumor stage, OR = 3.26, 95% CI: 2.13–4.98, *P* < 0.05; lymph node metastasis, in Asians, OR = 2.35, 95% CI: 1.53–3.60, *P* < 0.05; recurrence, OR = 10.71, 95% CI: 3.54–32.38, *P* < 0.05; T stage, in Asians, OR = 2.52, 95% CI: 1.66–3.83, *P* < 0.05). In addition, significant correlations between COX-2 overexpression and overall survival of laryngeal cancer were found both in Asians and in Caucasians (total, HR = 1.73, 95% CI: 1.23–2.24, *P* < 0.05; survival in Asians, HR = 2.59, 95% CI: 1.27–3.92, *P* < 0.05; survival in Caucasians, HR = 1.59, 95% CI: 1.03–2.14, *P* < 0.05).

**Conclusions:**

The meta-analysis results suggested that COX-2 overexpression was significantly associated with the increased risk, worse clinicopathological progression, and poorer prognosis of laryngeal cancer.

## Introduction

Laryngeal cancer is the second most common cancer of the upper respiratory tract, and it includes approximately 30% of all head and neck cancers (1). It was estimated that 13,000 new laryngeal cancer patients were diagnosed in the USA yearly, while approximately 25,300 new cases of laryngeal cancer were reported annually (2, 3). Although laryngectomy, radiotherapy, and chemotherapy were used to treat laryngeal cancer, the 5-year rate was still less than 30% (4). The selection of therapeutic methods depended on the tumor’s stage and sensitivity to chemoradiotherapy. In general, the larynx was sacrificed to obtain a better prognosis in the treatment of laryngeal cancer, which often affected physiological function and psychological health (1). To date, there were no effective biomarkers or tools to predict the progression and prognosis of laryngeal cancer, and almost 40% of cases were diagnosed as advanced stage tumors (5, 6). Therefore, studies were urgently conducted to discover effective biomarkers for the progression of laryngeal cancer and to elucidate the molecular mechanism of laryngeal carcinoma.

Cyclooxygenase-2 (COX-2) is one of the isoforms of cyclooxygenase, a membrane-bound and rate-limiting enzyme (7). COX-2 could catalyze the generation of prostaglandin E2 (PGE2), which is responsible for the normal physiological functions of human bodies (8). In general, COX-2 presents a lower expression level in normal tissues, and a higher expression of COX-2 is often found in many tumor tissues such as gastric cancer, breast cancer, endometrial cancer, and liver cancer (9–12). Considering the significant expression differences between normal tissues and cancer tissues, COX-2 might be a potential biomarker for the early diagnosis of tumors. The positive rate of COX-2 in lung cancer tissue was much higher than that in normal tissues (13). Furthermore, chemotherapy, radiotherapy, and proinflammatory cytokines promoted the expression of COX-2; thus, COX-2 might be related to the clinical progression of cancers (8). It has been reported that elevated COX-2 levels predicted poorer survival of non-small cell lung cancer (14), and higher COX-2 expression was significantly associated with histological type, lymph node metastasis, and venous invasion of liver cancer (15). Recently, many studies have been conducted to explore the correlation between the clinical progression of laryngeal cancer and COX-2 expression. However, the results were not consistent and convincing due to the sample size, source of the patients, and detection methods of COX-2 expression. Therefore, we carried out this meta-analysis to clarify the role of COX-2 overexpression in the risk, progression, and prognosis of laryngeal cancer.

## Methods

### Literature Search Strategy and Selection

PubMed, Embase, Web of Science, and CNKI were retrieved to search relevant literature in December 2021 with the following search terms: “COX-2”, “COX2”, “cyclooxygenase-2”, “expression”, “laryngeal cancer”, “laryngeal carcinoma”, and “prognosis”. Finally, relevant studies involving the associations of COX-2 expression with the risk, clinicopathological characteristics, and prognosis of laryngeal cancer were included.

### Inclusion and Exclusion Criteria

The inclusion criteria were as follows: 1) studies involving the associations of COX-2 expression with the risk, clinical characteristics, and prognosis of laryngeal cancer; 2) studies applying immunohistochemistry (IHC) to assess the expression of COX-2 in laryngeal tissue; 3) studies including enough data on the risk, clinical characteristics, and prognosis of laryngeal cancer to estimate OR, HR, and 95% CI; and 4) studies dividing COX-2 expression levels into positive and negative categories. The exclusion criteria included the following: 1) duplicate studies, letters, and reviews; 2) detection methods of COX-2 expression were not IHC; 3) studies conducted in cell lines and animal models; and 4) studies that did not offer enough data to calculate OR, HR, and 95% CI.

### Data Extraction and Quality Assessment

The following data were extracted from the eligible studies: “first authors’ name”, “publication year”, “patients’ country”, “cancer type”, “number of positive and negative cases of COX-2 in the case group and control group”, “detection method of COX-2 expression”, “cutoff value for COX-2 expression levels”, “overall survival curve for extracting HR and 95% CI”, and “HR and 95% CI data for overall survival of laryngeal cancer”. Two reviewers independently extracted the data from the included studies, and the two researchers discussed and resolved any discrepancies. To evaluate the methodological quality of eligible studies, the Newcastle-Ottawa Quality Assessment Scale (NOQAS) was used, and the included literature was scored from 0 to 9 (16).

### Statistical Analysis

Pooled OR and 95% CI were calculated and used to evaluate the associations of COX-2 overexpression with risk and clinical characteristics of laryngeal cancer. In comparison, HR and 95% CI were applied to assess the role of COX-2 overexpression in the overall survival of laryngeal cancer. Cochran’s *Q* statistic and *I*^2^ tests were used to assess the statistical heterogeneity among studies (17, 18). A random-effects model was used when the *I*^2^ value was >50% or *P <*0.05; when the *I*^2^ value was <50% or *P >*0.05, a fixed-effects model was applied (19). Begg’s and Egger’s tests were conducted to estimate publication bias (20, 21). Sensitivity analyses were also performed to observe the effects of individual studies on the overall results. If HR and 95% CI for laryngeal cancer survival were not directly provided, survival data would be extracted from the survival curve with Engauge Digitizer 11.1 software (22). All *P*-values were two-sided and *P <*0.05 was statistically significant. STATA 12.0 software (Stata Corporation, College Station, TX, USA) was used to carry out all the statistical analyses.

## Results

### Search Results and Study Characteristics

**Figure 1:**
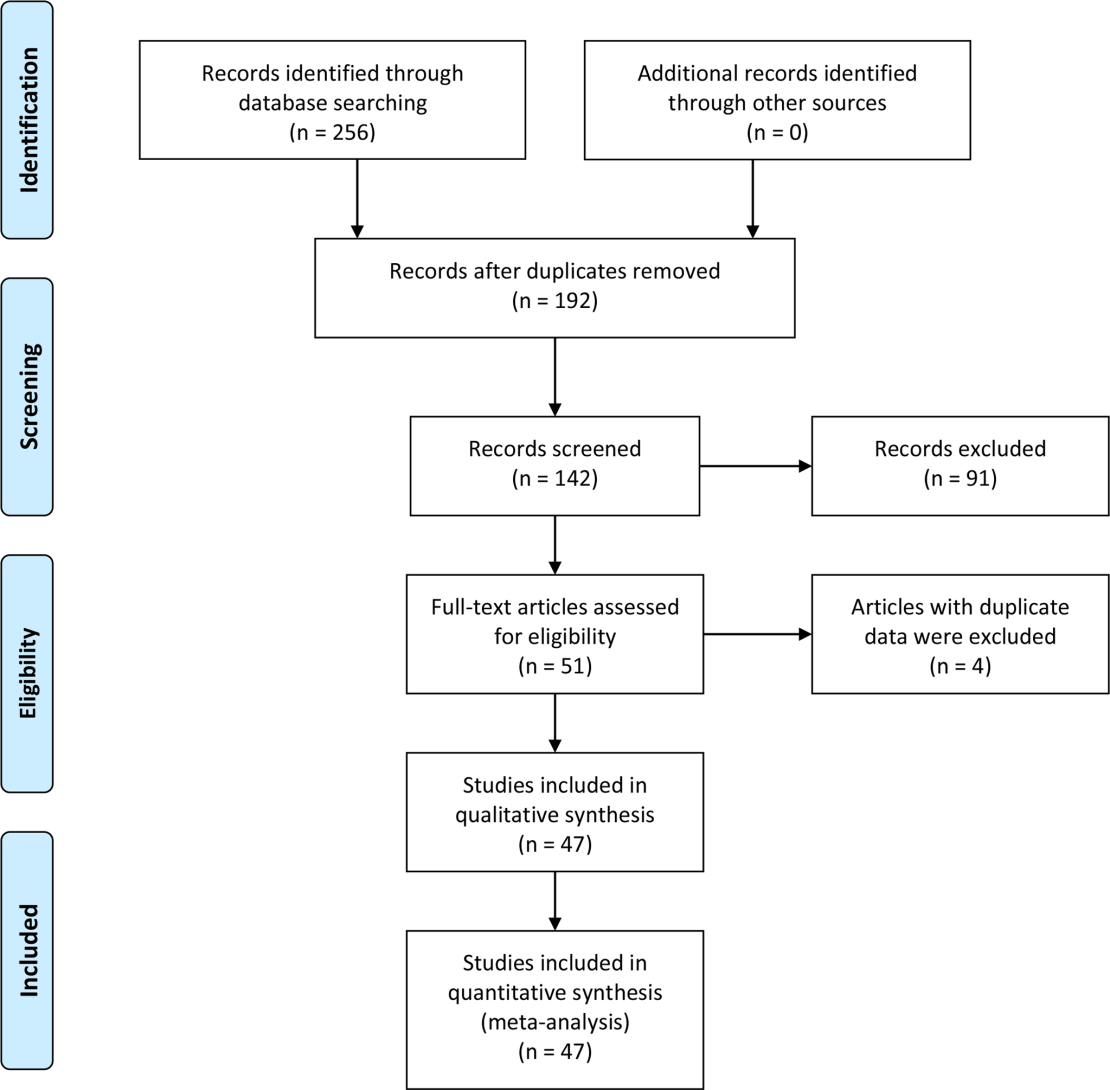
Flowchart of literature retrieval.

Two hundred fifty-six studies were acquired from an initial retrieval. Then, titles, abstracts, and full text were read in detail. Finally, according to the inclusion and exclusion criteria, 47 articles with 860 normal controls and 1,352 laryngeal cancer patients were identified (23–69). Twelve studies were conducted in Caucasians, and 35 reports were performed in Asians. In these included studies, 22 studies observed the correlation between COX-2 expression and risk of laryngeal cancer, and 46 reports involved the associations of COX-2 expression with clinical characteristics of laryngeal cancer. In addition, nine pieces of literature that explored the role of COX-2 in the overall survival of laryngeal cancer were included. The survival curve was extracted from five articles used to extract the HR and 95% CI, while HR and 95% CI data were extracted directly from the other four pieces of literature. The quality scores of eligible studies were all >6, which indicated the high quality of the included studies (**Figure 1** and **Tables 1**, **2**).

**Table 1:** Characteristics of the included studies for risk of laryngeal cancer patients.

Author	Reference	Time	Country	Ethnicity	Method	Disease type	Normal tissue		Tumor tissue		Cutoff value	NOS	
							COX-2^−^	COX-2^+^	COX-2^−^	COX-2^+^			
Xu	(23)	2004	China	Asians	IHC	Laryngeal cancer	22	0	22	44	10%	8
Jiang	(24)	2005	China	Asians	IHC	Laryngeal cancer	15	3	13	27	10%	8
Deng	(25)	2005	China	Asians	IHC	Laryngeal cancer	10	0	6	27	0%	7
Cho	(26)	2007	Korea	Asians	IHC	Laryngeal cancer	38	0	13	26	5%	8
Kourelis	(27)	2007	Greece	Caucasians	IHC	Laryngeal cancer	45	74	21	76	10%	7
Yin	(28)	2007	China	Asians	IHC	Laryngeal cancer	0	9	45	31	10%	8
Zhang	(29)	2007	China	Asians	IHC	Laryngeal cancer	7	0	23	34	5%	8
Huang	(30)	2007	China	Asians	IHC	Laryngeal cancer	13	4	9	37	5%	8
Chen	(31)	2008	China	Asians	IHC	Laryngeal cancer	81	0	42	39	None	8
Yao	(32)	2009	China	Asians	IHC	Laryngeal cancer	30	3	15	49	10%	7
Liang	(33)	2009	China	Asians	IHC	Laryngeal cancer	29	5	24	41	10%	8
Liu	(34)	2009	China	Asians	IHC	Laryngeal cancer	19	1	54	35	0%	7
Qin	(35)	2010	China	Asians	IHC	Laryngeal cancer	29	5	24	41	10%	8
Li	(36)	2011	China	Asians	IHC	Laryngeal cancer	36	14	19	31	10%	8
Liu	(37)	2012	China	Asians	IHC	Laryngeal cancer	27	7	18	47	None	8
Wang	(38)	2012	China	Asians	IHC	Laryngeal cancer	8	2	8	37	5%	7
Chen	(39)	2014	China	Asians	IHC	Laryngeal cancer	10	0	7	23	10%	7
Liu	(40)	2014	China	Asians	IHC	Laryngeal cancer	40	8	7	41	1%	8
Yao	(41)	2014	China	Asians	IHC	Laryngeal cancer	24	6	14	46	5%	8
Sun	(42)	2014	China	Asians	IHC	Laryngeal cancer	44	19	18	45	5%	8
Zeng	(43)	2016	China	Asians	IHC	Laryngeal cancer	68	30	30	68	10%	8
Zhu	(44)	2017	China	Asians	IHC	Laryngeal cancer	75	0	43	32	50%	8

IHC, immunohistochemistry; NOS, Newcastle-Ottawa Scale.

**Table 2:** Characteristics of the included studies for overall survival of laryngeal cancer patients.

Author	Reference	Time	Country	Ethnicity	Disease type	No.	Follow-up (months)	Method	Source of HR	HR	95% CI	*P*	Cutoff
Liang	(33)	2009	China	Asians	Laryngeal cancer	65	0–90	IHC	Curve	2.75	1.73–15.09	<0.05	10%
Qin	(35)	2010	China	Asians	Laryngeal cancer	65	0–100	IHC	Curve	1.75	1.28–5.37	<0.05	10%
Ranelletti	(45)	2001	Italy	Caucasians	Laryngeal cancer	61	None	IHC	Survival data	12.80	1.63–101.4	0.015	None
Perezruiz	(46)	2012	Spain	Caucasians	Laryngeal cancer	59	0–150	IHC	Curve	1.30	0.57–2.96	>0.05	10%
Chen	(53)	2013	China	Asians	Laryngeal cancer	80	0–200	IHC	Survival data	11.164	4.628–26.93	<0.05	10%
Dong	(58)	2007	China	Asians	Laryngeal cancer	68	0–40	IHC	Curve	0.45	0.24–0.98	0.02	5%
Sackett	(67)	2008	Canada	Caucasians	Laryngeal cancer	201	0–120	IHC	Survival data	1.62	1.04–2.53	0.04	50%
Cho	(68)	2004	USA	Caucasians	Laryngeal cancer	105	0–120	IHC	Survival data	2.57	1.21–5.47	0.01	0%
Hoing	(69)	2018	Germany	Caucasians	Laryngeal cancer	101	0–40	IHC	Curve	0.44	0.17–0.85	0.04	None

IHC, immunohistochemistry; NOS, Newcastle-Ottawa Scale; HR, hazard ratio; CI, confidence interval.

### Meta-Analysis Results

**Figure 2:**
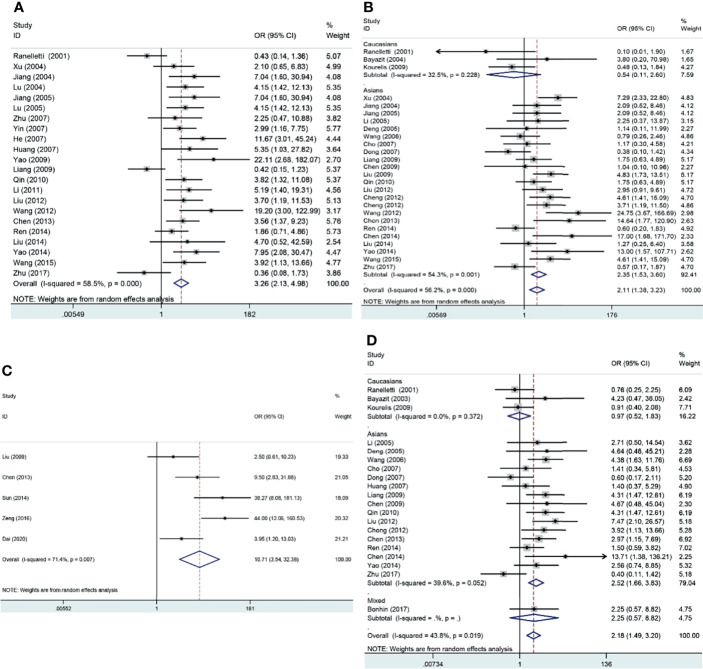
Forest plots of the associations of COX-2 overexpression with clinical features of laryngeal cancer. **(A)** Tumor stage for laryngeal cancer. **(B)** Lymph node metastasis for laryngeal cancer. **(C)** Recurrence for laryngeal cancer. **(D)** T stage for laryngeal cancer. OR, odds ratio; CI, confidence interval.

The pooled results suggested that COX-2 overexpression was significantly associated with the risk of laryngeal cancer (COX-2 positive in laryngeal cancer vs. COX-2 positive in normal control: 64.87% vs. 22.09%; OR = 11.62, 95% CI: 6.96–19.40, *P* < 0.05). However, a small heterogeneity was found in the analysis for the risk of laryngeal cancer (*I*^2^ = 69.1%, *P* < 0.001), in which the type of antibodies, experimental methods of IHC, and cutoff values for evaluating COX-2 expression might lead to the heterogeneity. In addition, we found that COX-2 overexpression was significantly associated with the tumor stage and T stage of laryngeal cancer (tumor stage, OR = 3.26, 95% CI: 2.13–4.98, *P* < 0.05; recurrence, OR = 10.71, 95% CI: 3.54–32.38, *P* < 0.05). Subgroup analysis suggested that there was a significant association of COX-2 overexpression with the lymph node metastasis and T stage of laryngeal cancer (lymph node metastasis, total, OR = 2.11, 95% CI: 1.38–3.23, *P* < 0.05, in Asians, OR = 2.35, 95% CI: 1.53–3.60, *P* < 0.05; T stage, total, OR = 2.18, 95% CI: 1.49–3.20, *P* < 0.05, in Asians, OR = 2.52, 95% CI: 1.66–3.83, *P* < 0.05), and heterogeneity among studies was significantly decreased in the subgroup analysis based on ethnicity. Finally, we estimated the prognostic value of COX-2 overexpression in laryngeal cancer, and significant correlations between COX-2 overexpression and poor prognosis of laryngeal cancer were found both in Asians and Caucasians (total, HR = 1.73, 95% CI: 1.23–2.24; survival in Asians, HR = 2.59, 95% CI: 1.27–3.92, *P* < 0.05; survival in Caucasians, HR = 1.59, 95% CI: 1.03–2.14, *P* < 0.05) (**Figure 2** and **Table 3**). 

**Table 3:** Meta-analysis results for COX-2 overexpression in risk, clinical features, and overall survival of laryngeal cancer patients.

**Characteristics**	**Studies**	**Number of cases**	**Pooled OR (95% CI)**	***P***	**Heterogeneity**		**Begg’s test**		**Egger’s test**	
					***I*^2^ (%)**	***P***	***Z***	***P***	***T***	***P***
Risk	(21)	1,352	11.62 (6.96–19.40)	<0.05	69.1	<0.001	2.11	0.03	3.26	0.004
Tumor grade	(6)	432	0.75 (0.29–1.93)	>0.05	74.3	0.001	0.30	0.76	0.04	0.97
Caucasian	(3)	217	0.52 (0.14–1.94)	>0.05	77.2	0.012	0.00	1	0.08	0.95
Asian	(4)	215	1.01 (0.24–4.27)	>0.05	74.1	0.009	0.34	0.73	−1.35	0.31
Tumor stage	(21)	1,309	3.26 (2.13–4.98)	<0.05	58.5	<0.001	2.23	0.03	1.68	0.11
Lymph node metastasis	(25)	1,566	2.11 (1.38–3.23)	<0.05	56.2	0.001	0.51	0.61	0.53	0.60
Caucasian	(3)	230	0.54 (0.11–2.60)	>0.05	32.5	0.228	0.00	1	0.13	0.92
Asian	(22)	1,336	2.35 (1.53–3.60)	<0.05	54.3	0.001	0.77	0.44	1.20	0.24
Recurrence	(1)	384	10.71 (3.54–32.38)	<0.05	64.3	0.015	0.24	0.81	0.60	0.59
Glottic cancer	(10)	628	1.10 (0.76–21.59)	>0.05	24.7	0.209	0.54	0.59	0.20	0.84
Caucasian	(4)	185	1.14 (0.59–2.20)	>0.05	55.1	0.083	0.34	0.73	−0.07	0.95
Asian	(6)	443	1.08 (0.69–1.68)	>0.05	8.7	0.362	1.05	0.29	0.63	0.56
T stage	(18)	1,191	2.18 (1.49–3.20)	<0.05	43.8	0.019	0.78	0.44	1.28	0.22
Caucasian	(3)	230	0.97 (0.52–1.83)	>0.05	0	0.372	0.00	1.00	1.75	0.33
Asian	(15)	961	2.52 (1.66–3.83)	<0.05	39.6	0.052	0.16	0.87	0.46	0.65
Differentiation	(22)	1,541	1.56 (0.97–2.52)	>0.05	72.1	<0.001	0.87	0.38	1.22	0.24
Gender	(12)	792	1.23 (0.82–1.84)	>0.05	0	0.899	0.24	0.81	−0.62	0.55
Age	(9)	1,176	1.00 (0.77–1.28)	>0.05	0	0.947	1.97	0.05	0.11	0.92
Caucasian	(3)	735	1.02 (0.75–1.37)	>0.05	0	0.375	0	1.00	−0.29	0.82
Asian	(6)	441	0.95 (0.61–1.48)	>0.05	0	0.968	2.10	0.04	4.04	0.01
			Pooled HR (95% CI)							
Overall survival	(8)	805	1.73 (1.23–2.24)	<0.05	0	0.615	1.56	0.12	1.7	0.13
Caucasian	(1)	527	1.59 (1.03–2.14)	<0.05	0	0.863	0.24	0.806	1.85	0.16
Asian	(4)	278	2.59 (1.27–3.92)	<0.05	4	0.373	0.34	0.734	0.48	0.68

OR, odds ratio; HR, hazard ratio; CI, confidence interval.

### Publication Bias and Sensitivity Analysis

**Figure 3:**
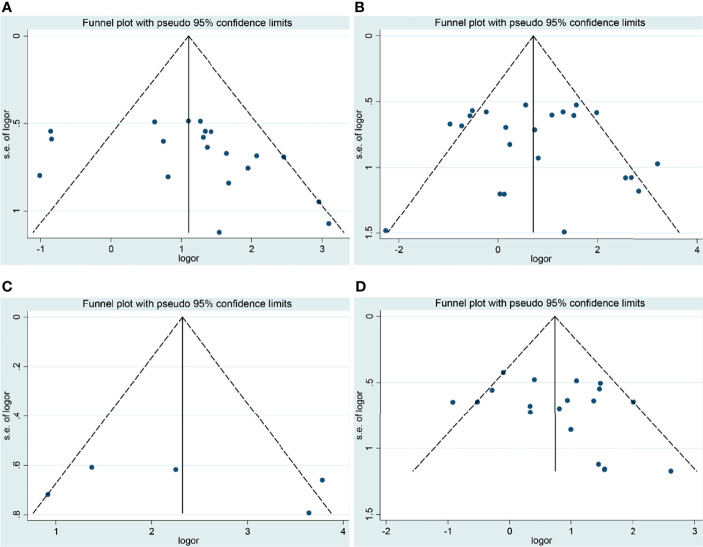
Funnel plots of the association of COX-2 expression with clinical features of laryngeal cancer. **(A)** Tumor stage for laryngeal cancer. **(B)** Lymph node metastasis for laryngeal cancer. **(C)** Recurrence for laryngeal cancer. **(D)** T stage for laryngeal cancer. OR, odds ratio; CI, confidence interval.

**Figure 4:**
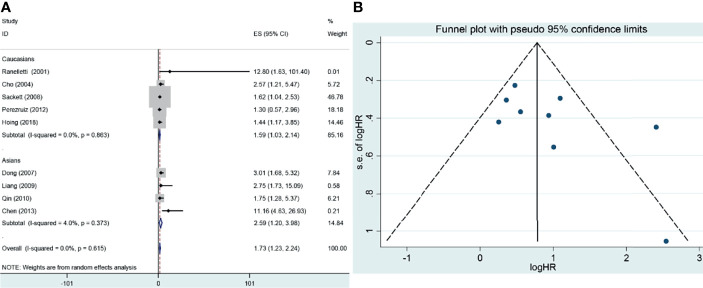
Forest plot and funnel plot of the association between COX-2 expression and overall survival of laryngeal cancer. **(A)** Forest plot for laryngeal cancer. **(B)** Funnel plot for laryngeal cancer. HR, hazard ratio; CI, confidence interval.

Although a small heterogeneity was found, sensitivity analysis did not identify any studies that significantly affected the overall statistical results. According to Begg’s test and Egger’s test, publication bias was found in the analysis for the risk and tumor stage of laryngeal cancer. The studies were mainly conducted in the Chinese population. Moreover, we speculated that antibody differences, experimental methods of IHC, evaluation methods of COX-2 expression, and cutoff values for COX-2 expression might contribute to the slight publication bias (**Figures 3**, **4**).

## Discussion

The main risk factors for laryngeal cancer were tobacco use, alcohol consumption, and human papillomavirus infection. However, recent studies have found numerous genetic alterations in laryngeal cancer, which suggested that genetics also was involved in the occurrence of laryngeal cancer. In a genome-wide association study involving 993 laryngeal squamous cell carcinoma patients and 1,995 controls, researchers found the three most significant SNPs—rs174549, rs2857595, and rs10492336—which are located in FASD1, AIF1, and TBX5 genes, respectively (70). In addition, a gene expression study conducted in laryngeal cancer tissues and adjacent normal tissues suggested that differentially expressed genes were mainly enriched in cell cycle, DNA replication, metabolic pathways, mucin-type O-glycan biosynthesis, and drug metabolism-cytochrome P450 (71). Therefore, the internal genetic mechanism might be responsible for laryngeal cancer’s occurrence and clinical progression. In addition to the high-throughput studies, some articles were also conducted to detect the expression of biomarkers in scattered tumor tissues. In these biomarkers, COX-2 was involved in inflammation, cellular invasion, angiogenesis, antiapoptotic cellular defenses, and immunological resistance (72).

In the present study, we found that people with elevated COX-2 expression had a higher risk for laryngeal cancer, in which only one study was conducted in Caucasians (27). Thus, the conclusion might be more appropriate to Asians, and more Caucasians should be included in future studies. Moreover, a small heterogeneity was detected in the analysis for the risk of laryngeal cancer, but sensitivity analysis found no significant difference. Moreover, COX-2 expression was significantly associated with the stage and recurrence of laryngeal cancer, in which the included subjects were mostly from China, except for one study that involved participants from Italy. The subgroup analysis found that COX-2 expression had a significant association with lymph node metastasis and T stage of laryngeal cancer in Asians but not in Caucasians. We also conducted a correlation analysis between COX-2 overexpression and tumor grade, tumor type (glottic cancer vs. non-glottic cancer), differentiation, gender, and age (<60 vs. >60). However, no significant associations were found.

To observe the role of COX-2 overexpression in the prognosis of laryngeal cancer, nine studies were included, of which four were from Caucasians and five were from Asians. In the overall analysis, there was a significant association between COX-2 overexpression and overall survival of laryngeal cancer (HR = 1.73, 95% CI: 1.23–2.24, *P* < 0.05). A significant association was also found in the subgroup analysis based on ethnicity (survival in Asians, HR = 2.59, 95% CI: 1.27–3.92, *P* < 0.05; survival in Caucasians, HR = 1.59, 95% CI: 1.03–2.14, *P* < 0.05). In the nine eligible studies, Perezruiz et al. got a negative result, and Hoing et al. obtained an opposite result (46, 69). Sensitivity analysis suggested that these studies did not affect the pooled overall results, and no significant heterogeneity and publication bias were found in the analysis for the overall survival of laryngeal cancer, suggesting that the results were stable.

According to the study results, we speculated that COX-2 overexpression was a clinical biomarker for laryngeal cancer that might affect the clinical progression and prognosis of laryngeal cancer. Although this was the first meta-analysis to assess the association of COX-2 overexpression with laryngeal cancer, some limitations should be addressed. Firstly, the included studies were primarily performed to explore the role of COX-2 expression in the risk, tumor stage, lymph node metastasis, recurrence, differentiation, and gender of laryngeal cancer in Asians. Secondly, the cutoff values of COX-2 expression were not unified, leading to heterogeneity among studies. Thirdly, the overall survival data were extracted from survival curves rather than original variance data, which might lead to the deviation of the final results. Fourthly, the study’s sample size was still too small after performing a subgroup analysis.

In conclusion, COX-2 overexpression was significantly associated with the higher risk and worse prognosis of laryngeal cancer. Moreover, COX-2 overexpression had significant associations with the tumor stage, lymph node metastasis, recurrence, and T stage of laryngeal cancer. Finally, more studies on the correlations of COX-2 overexpression with the risk, clinical characteristics, and prognosis of laryngeal cancer should be performed in the future, especially in Caucasians.

## Data Availability Statement

The datasets presented in this study can be found in online repositories. The names of the repository/repositories and accession number(s) can be found in the article/supplementary material.

## Author Contributions

All authors listed have made a substantial, direct, and intellectual contribution to the work and approved it for publication.

## Funding

The study was supported by “Nanchong 2020 City-School Science and Technology Strategic Cooperation Special Project (North Sichuan Medical College) (No. 20SXQT0105)”, “Nanchong 2019 City-School Cooperation Scientific Research Special Project (North Sichuan Medical College) (No. 19SXHZ0130)” and “Science Research Project of Sichuan Provincial Department of Education (No. 15ZB0188)”.

## Conflict of Interest

The authors declare that the research was conducted in the absence of any commercial or financial relationships that could be construed as a potential conflict of interest.

## Publisher’s Note

All claims expressed in this article are solely those of the authors and do not necessarily represent those of their affiliated organizations, or those of the publisher, the editors and the reviewers. Any product that may be evaluated in this article, or claim that may be made by its manufacturer, is not guaranteed or endorsed by the publisher.
